# Inconsistent Effects of Glatiramer Acetate Treatment in the 5xFAD Mouse Model of Alzheimer’s Disease

**DOI:** 10.3390/pharmaceutics15071809

**Published:** 2023-06-24

**Authors:** Berke Karaahmet, John A. Olschowka, M. Kerry O’Banion

**Affiliations:** 1 Department of Neuroscience, Del Monte Institute for Neuroscience, University of Rochester Medical Center, Rochester, NY 14642, USA; bk2895@cumc.columbia.edu (B.K.); john_olschowka@urmc.rochester.edu (J.A.O.); 2 Department of Neurology, University of Rochester Medical Center, Rochester, NY 14642, USA

**Keywords:** microglia, Alzheimer’s disease, glatiramer acetate

## Abstract

Alzheimer’s disease (AD) is a chronic neurodegenerative disorder that involves strong inflammatory components. Aberrant and prolonged inflammation in the CNS is thought to contribute to the development of the pathology. The use of single cytokine approaches to curb or leverage inflammatory mechanisms for disease modifying benefit has often resulted in conflicting data. Furthermore, these treatments were usually delivered locally into the CNS parenchyma, complicating translational efforts. To overcome these hurdles, we tested the use of glatiramer acetate (GA) in reducing amyloid beta (Aβ) plaque pathology in the 5xFAD model of AD. GA immunizations were begun at the ages of 2.5 months, 5.5 months, and 8.5 months, and GA was delivered weekly for 8 weeks. While previous data describe potential benefits of GA immunization in decreasing Aβ levels in murine models of AD, we found modest decreases in Aβ levels if given during the development of pathology but, surprisingly, found increased Aβ levels if GA was administered at later stages. The impact of GA treatment was only significant for female mice. Furthermore, we observed no changes between microglial uptake of plaque, CD11c immunopositivity of microglia, or levels of TMEM119 and P2Ry12 on microglia. Overall, these data warrant exercising caution when aiming to repurpose GA for AD.

## 1. Introduction

Alzheimer’s disease is a neurodegenerative disorder characterized by the accumulation of extracellular amyloid beta (Aβ) and intracellular neurofibrillary tangles composed of hyperphosphorylated tau. The leading hypothesis in the pathogenesis of AD, the amyloid cascade hypothesis, implicates aberrant inflammation of the CNS, driven by Aβ accumulation, as the central link between these two molecular hallmarks of the disease [1]. The complex interplay between Aβ, inflammation and tau hyperphosphorylation ultimately leads to neuronal dysfunction and clinical dementia [2].

Microglia, the immunocompetent resident macrophages of the CNS, are one of the earliest responders to AD neuropathology. They perform key phagocytic and enzymatic degradation functions under homeostatic conditions, but the chronic inflammatory environment associated with AD impairs these functions [3,4]. A number of studies have attempted to modulate the activation profiles of microglia by using intracerebrally administered or virally expressed cytokines of a pro- or anti-inflammatory nature [5,6,7,8,9]; reviewed in [10]. Conflicting data from these studies, as well as increasing appreciation for microglial heterogeneity [11], has encouraged investigations of broad immunomodulatory treatments [10]. Furthermore, modulation of peripheral inflammation has been observed to impact microglial activity and ultimately, the magnitude of AD-like pathology [12,13,14]. Altogether, these data provide the impetus for investigation of peripherally administered, immunomodulatory therapeutics for AD.

Glatiramer acetate (GA) is a promising candidate to modulate CNS inflammation. GA, also known as copolymer 1 or Copaxone, is a mixture of random salts of synthetic polypeptides containing L-alanine, L-glutamate, L-lysine, and L-tyrosine [15]. This mixture of random length polypeptides is thought to mimic myelin antigens since the four amino acids of GA are enriched in myelin basic protein (MBP). Although historically designed to induce experimental autoimmune encephalomyelitis (EAE), GA was shown to be protective against it [16]. In the clinic, GA is still used to prevent relapsing remitting multiple sclerosis. While the mechanism of action of GA is unclear, the most popular hypotheses in EAE models involve the induction of myelin-reactive, anti-inflammatory Th2 cells which limit reactive autoaggression of Th1/Th17 cells or promotion of type II monocytes, which are characterized by increased secretion of IL-10 and TGF-β [17,18,19,20,21].

The hypothesis that GA may have anti-inflammatory properties led to investigations of its therapeutic potential in various AD-like mouse models. Early reports in the APP/PS1 model showed that passive weekly GA vaccination can reduce Aβ accumulation and that this effect correlated with CD11c^+^ microglia-shaped cells [22]. Recent data has suggested that CD11c^+^ microglia may represent the disease-associated microglia (DAM) phenotype which could be beneficial in AD-like pathology [23,24]. Further reports showed that GA is effective even in 18-month-old APP/PS1 mice [25]. At a cellular level, GA is partly thought to increase the expression of osteopontin on peripheral monocytes which infiltrate the brain tissue and are home to Aβ plaques [26]. Recent work from our laboratory showed that GA was also effective in reducing amyloid pathology and modifying tau hyperphosphorylation states in the 3xTg model. However, in our experiments we did not identify increased infiltration of CD11b^+^CD45^hi^ monocytes [27]. RNAseq of purified microglia populations revealed only two differentially regulated genes, which raises questions on the impact of GA on microglia [27].

In this study, we investigated the impact of GA in both sexes of 5xFAD mice of various ages. In contrast to previous reports which observed significant reductions in plaque load [28], we saw no decrease in 2.5-month-old 5xFAD animals that were treated for 8 weeks with GA. This 8-week-long treatment had also previously shown benefits in our work and that of others using other models of AD [27,29]. Using the same weekly, 8-week long, subcutaneous GA treatment paradigm, we saw modest, insignificant reductions in plaque load in female 5.5-month-old 5xFAD mice. Surprisingly, the plaque load increased in female mice if the GA treatment was started at 8.5 months of age. The male mice showed no change in pathology in response to GA at any of these timepoints. Furthermore, we failed to identify (i) a discernable population of infiltrating monocytes, (ii) any meaningful changes in microglial expression of homeostatic markers P2Ry12 and TMEM119, (iii) microglial plaque uptake, or (iv) microglial expression of CD11c as determined by flow cytometry.

The implications of this work are two-fold: first, caution must be exercised when attempting to repurpose GA for late-stage AD and that GA may be more effective in females than in males; second, the cellular mechanisms of GA may involve mechanisms other than the manipulation of microglial activity, such as T-cell differentiation mechanisms or modulation of neuronal homeostasis and neurogenesis [19,22].

## 2. Materials and Methods

### 2.1. Transgenic Mice

All animal procedures were reviewed and approved by the University Committee on Animal Resources of the University of Rochester Medical Center for compliance with federal regulations prior to the initiation of the study. Hemizygous B6SJL-Tg(APPSwFlLon, PSEN1*M146L*L286V)6799Vas/Mmjax mice (abbreviated as 5xFAD) were used in this study. These 5xFAD mice overexpress amyloid precursor protein with Swedish, Florida, and London mutations along with M146L and L286V mutations in presenilin 1 [30]. This mouse model starts accumulating plaque around 2 months of age and the plaque load progressively and rapidly increases thereafter [30,31]. Non-transgenic wild-type (wt) litter-mates were used as controls where applicable. We used both sexes and we chose 3 timepoints at which to begin GA treatment: 2.5, 5.5, and 8.5 months of age.

### 2.2. GA Immunization

The 5xFAD mice received subcutaneous injections of GA (ApexBio) at 500 ng/μL in phosphate-buffered saline (PBS) or PBS alone (control group) twice during the first week of treatment and then once a week for 7 additional weeks. Each mouse received 200 μL per injection. This treatment regimen was selected based on previous studies with GA in other AD-like models [22,27,28,29]. The mice were weighed prior to initiation of the treatment and weekly thereafter.

### 2.3. Flow Cytometry/FACS

The mice were injected 24 h before sacrifice with Methoxy-X04 (MeX04, i.p., 4 mg/kg, Tocris Biosciences, Minneapolis, MN, USA), a brain-permeable Aβ fluorescent marker [32,33]. On the day of sacrifice, the animals were deeply anesthetized with a mixture of xylazine (i.p., 10 mg/kg) and ketamine (i.p., 100 mg/kg) and perfused intracardially with 0.15 M phosphate buffer (PB) containing 0.5% sodium nitrite and 2 IU heparin/mL. After perfusion, the hemispheres were separated: one was either immediately submerged in fixative solution (4% paraformaldehyde (PFA), pH 7.2 in PB, 4 °C) to be used for immunofluorescence experiments or flash-frozen in cold isopentane for ELISA (both as described below), and the other was processed for flow cytometry as follows. The hippocampus from each half-brain was dissected and homogenized in 3 mL FACS buffer (1× phosphate-buffered saline (PBS) + 0.5% BSA). Homogenates were filtered through a 70 µm cell strainer into a 15 mL tube containing 3 mL FACS buffer. The strainer was washed with an additional 3 mL of FACS buffer, and the cell suspensions were centrifuged at 400× *g* for 5 min at 4 °C. The supernatants were discarded, and the remaining pellets were resuspended in 40% percoll (Cytiva, Marlborough, MA, USA) prepared with PBS, then centrifuged at 400× *g* for 30 min with no braking. After removing the supernatants, the pellets were resuspended in 90 µL FACS buffer with 1:100 Fc block (2.4G2, 1:100, BioLegend, San Diego, CA, USA) and transferred to a 96-well plate. After a 15 min incubation with Fc block at 4 °C, the following antibodies were added in a 10 µL master mix: CD11b-FITC (M1/70, BioLegend), CD45-APC/Cy7 (30F11, BioLegend), CD11c-PE/Cy7 (N418, BioLegend), P2Ry12-APC (S16007D, BioLegend), and TMEM119-PE (106-6, Abcam, Boston, MA, USA). The latter two cell surface molecules are considered homeostatic microglial markers [23]. The plate was then incubated for 30 min at 4 °C in the dark. The samples were washed once with FACS buffer and transferred to 5 mL tubes containing 7AAD, a live/dead stain, such that its final dilution was 1:80. Appropriate fluorescent-minus-one (FMO) and single-stained bead controls (Ultracomp eBeads, Invitrogen, Waltham, MA, USA) were prepared in tandem with the samples. After excluding debris, doublets, and dead cells, CD45^int^/CD11b^+^ was used to gate and sort for microglia on a FACSAria II (BD). All events were recorded, and data were analyzed with FCS Express 7 (DeNovo Software, Pasadena, CA, USA).

### 2.4. Immunofluorescence

The half-brains were fixed overnight, equilibrated in 30% sucrose in PB overnight, frozen in cold isopentane, and stored at −80 °C. The frozen brains were then cryosectioned into 30 μm sections on a −25 °C freezing stage microtome and free-floating sections were stored in a cryoprotectant solution until assayed. For immunofluorescence protocols, sections were washed to remove the cryoprotectant, blocked with 10% normal goat serum, and incubated in primary antibody for 48 h at 4 °C. For Aβ immunostaining, sections were incubated with 70% formic acid for 3 min after the first wash and washed prior to the blocking step. After primary antibody incubation, the sections were washed and incubated with secondary antibodies bound to Alexa fluorophores (Invitrogen) for 2 h at room temperature. Sections were mounted and cover slipped with Prolong Gold (Thermo Fisher, Waltham, MA, USA). Primary and secondary antibody dilutions were as follows: biotinylated 6E10 (BioLegend #803008) 1:2000; rabbit anti-Iba1 (Wako #019-19741) 1:3000; rat anti-LAMP1 (eBioscience #14-1071-82) 1:2000; rat anti-mDectin-1 (CLEC7A, InvivoGen #mabg-mdect, San Diego, CA, USA) 1:800; goat anti-rat IgG Alexa 488 (Invitrogen #A-11006) 1:1000; goat anti-rabbit IgG Alexa 647 (Invitrogen #A-21245) 1:1500, and Streptavidin Alexa 594 or 488 (Invitrogen #S11227) 1:1000.

### 2.5. Image Acquisition and Analysis

For each animal, 2–3 coronal tissue sections that included the subiculum, CA1 field of the hippocampus (CA1), and dentate gyrus were imaged with a Nikon A1R HD confocal microscope using a 10× (Plan Apo Lambda, NA: 0.40) or 20× (Plan Apo VC, NA: 0.75) objective lens, as indicated in the figure legends. The imaging parameters were kept constant across all sections for each set of immunofluorescent experiments. All image analysis was performed using the ImageJ FIJI (NIH) software with semi-automated custom macros. The experimenters were blinded to the treatment.

For area fraction analysis of 6E10 (Aβ), MeX04 (core Aβ), and LAMP1, regions of interest (ROIs) outlining the subiculum, CA1, and dentate gyrus with CA4 were drawn on maximum z-projections of the acquired LAMP1 images. The images were subsequently thresholded and binarized using ImageJ’s automated Otsu thresholding algorithm, which was used for all other thresholding steps in this manuscript (except for MeX04 analysis, for which MaxEntropy was used). The plaque area fraction was calculated automatically by ImageJ. The overlap of 6E10^+^ Aβ plaque and Iba1^+^ or CLEC7A^+^ microglia was calculated per stack by enlarging the Otsu-thresholded plaques by approximately 8 μm and overlaying the mask on the Otsu-thresholded microglia image. The 6E10 masks and their microglial coverage areas were summed across stacks and their ratios over the sections were averaged to yield the percentage of plaque area covered by microglia. Plaques < 10 μm^2^ were not included in this analysis.

### 2.6. Statistics

Observers were blinded to subject treatment prior to immunohistochemistry analysis. All statistical comparisons were performed using Prism 7.0 (Graphpad Software, Boston, MA, USA). A *p*-value ≤ 0.05 was considered significant. Data in which two categorical variables were compared were analyzed with two-way analysis of variance (ANOVA). A Tukey’s multiple comparison was used to establish significance between individual groups. Student’s *t*-test or the Mann–Whitney test were employed when two group means were compared.

## 3. Results

### 3.1. GA Treatment Has Differential Effects on Amyloid Plaque Accumulation in Female 5xFAD Mice Depending on Age

**Figure 1:**
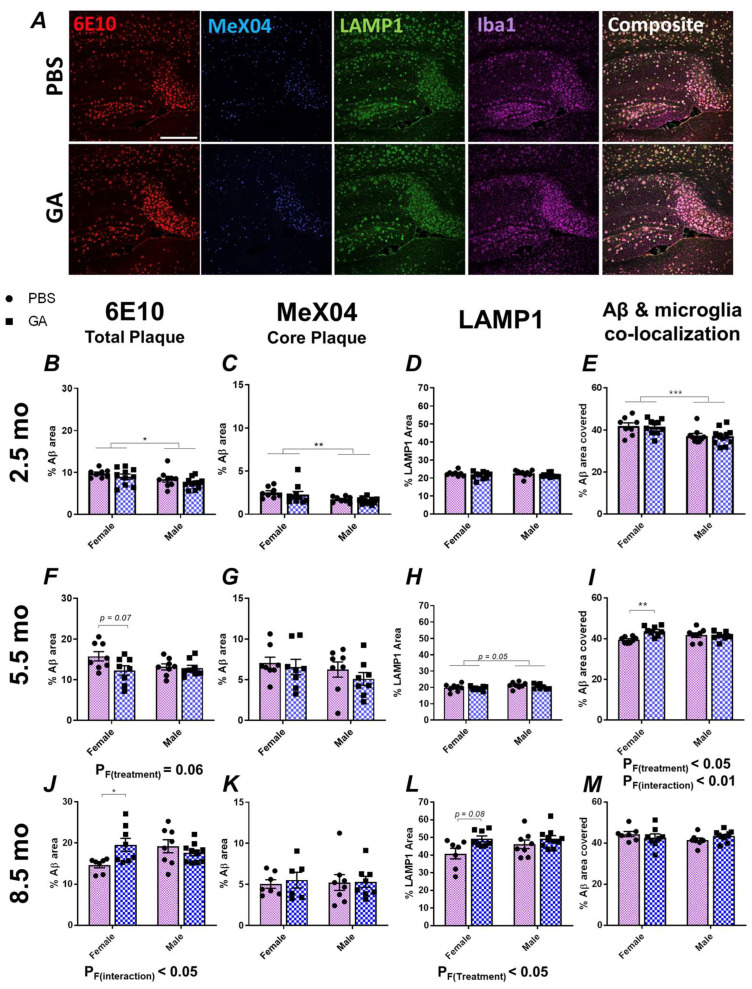
GA treatment shows differential impact on plaque pathology in the subiculum of female 5xFAD mice. Representative 10× images of 6E10^+^ Aβ (red), MeX04^+^ core plaques (blue), LAMP1^+^ dystrophic neurites (green), and Iba1^+^ microglia (purple) (**A**). Scale bar represents 500 μm. We assessed the percentage of area covered by 6E10^+^ Aβ plaques after 2 months of GA treatment beginning at 2.5 (**B**), 5.5 (**F**), or 8.5 months (**J**) of age. GA treatment led to a trend towards decreased 6E10 immunopositivity in females at the 5.5-month timepoint (**F**) but significantly increased 6E10 immunopositivity at the 8.5-month timepoint (**J**). Percentage of MeX04^+^ plaque area was not affected by GA treatment at the 2.5 (**C**), 5.5 (**G**), or 8.5-month timepoints (**K**). Percentage of LAMP1^+^ area was not significantly affected by GA treatment at the 2.5 (**D**) and 5.5-month (**H**) timepoints. There was a significant effect of GA treatment on LAMP1 immunopositivity at the 8.5-month timepoint and post hoc analyses showed a trend towards increased LAMP1 levels in GA-treated females (**L**). Quantification of 6E10^+^ area covered by Iba1^+^ microglia showed no effect of GA treatment at the 2.5 (**E**) and 8.5-month (**M**) timepoints. Microglial colocalization was significantly increased at 5.5-month timepoint in GA-treated female mice compared to controls (**I**). Numerical data represented as mean ± SEM using circles and pink boxes for PBS treatment and squares and blue boxes for GA treatment. *n* = 7–10 animals per group. * *p* < 0.05, ** *p* < 0.01, *** *p* < 0.001. Two-way ANOVA with post hoc multiple comparisons.

Previous data, including those from our past work, have shown that GA effectively reduces the plaque load or Aβ levels in APP/PS1, 5xFAD and 3xTg models of AD [22,26,27,28,29]. In the present study, we aimed to replicate some of these findings in 2.5-month-old 5xFAD mice (Figure 1). In contrast to published data, we saw no changes in Aβ pathology as quantified by 6E10 and MeX04 area in the subiculum of both sexes (Figure 1B,C). Similarly, there was no significant effect of GA treatment in neuritic damage, as quantified by LAMP1 immunopositivity or microglial colocalization with 6E10^+^ Aβ plaques (Figure 1D,E).

These unexpected findings prompted us to test two additional cohorts: a 5.5-month-old cohort, in which the treatment was initiated during the developing plaque pathology, and an 8.5-month-old cohort, in which the treatment was initiated after extensive amyloid deposition [30]. In the 5.5-month-old cohort, analysis of the 6E10^+^ Aβ plaques revealed a trend towards reduced plaque following GA treatment in female mice (Figure 1F, *p* _F(Treatment)_ = 0.06, post hoc *p* _(PBS vs. GA)_ = 0.07) but not in male mice. On the other hand, in the 8.5-month-old cohort, analysis of the 6E10^+^ Aβ plaque area revealed a significant effect of GA treatment (*p* _F(Treatment)_ < 0.05) and post hoc analysis showed significantly increased Aβ levels (*p* < 0.05) only in the female mice (Figure 1J). There was no significant effect of GA treatment on the MeX04^+^ plaques (Figure 1G,K). Consistent with the above, we saw a trend towards an increase in LAMP1^+^ dystrophic neurites in female GA-treated mice of the 8.5-month-old cohort compared to PBS controls (Figure 1L, *p* = 0.08) and a significant increase in Iba1^+^ microglial colocalization with Aβ plaques in GA-treated female mice of the 5.5-month-old cohort compared to PBS controls (Figure, 1I, *p* _(Treatment x Sex)_ < 0.01). There was no significant effect of GA on Iba1^+^ microglial colocalization with plaque in the 8.5-month-old cohort (Figure 1M) or LAMP1 immunopositivity in the 5.5-month-old cohort (Figure 1H). The body weights of the mice did not change significantly compared to the PBS controls at any of these timepoints, suggesting that GA was well-tolerated (Supplementary Figure S1).

We chose to focus on the subiculum because our previous data in 3xTg mice showed a reduction in the plaque load in this region, and the subiculum displays the highest plaque pathology in 5xFAD mice. We extended our analyses to regions of interest involving CA1 and dentate gyrus with CA4 (DG) (Supplementary Figures S2 and S3). While we observed some trends that mirrored our findings in the subiculum, such as a trend towards increased plaque (Supplementary Figure S3) and significantly reduced microglia coverage of Aβ in the DG of 8.5-month-old female 5xFAD mice (Supplementary Figure S3), we did not find any significant effect of GA treatment in our comparisons in CA1 or DG (Supplementary Figures S2 and S3). This may be due to the increased variability from having less plaque present in these regions compared to the subiculum.

### 3.2. GA Treatment Does Not Change Surface Levels of Microglial P2Ry12, TMEM119, or CD11c

**Figure 2:**
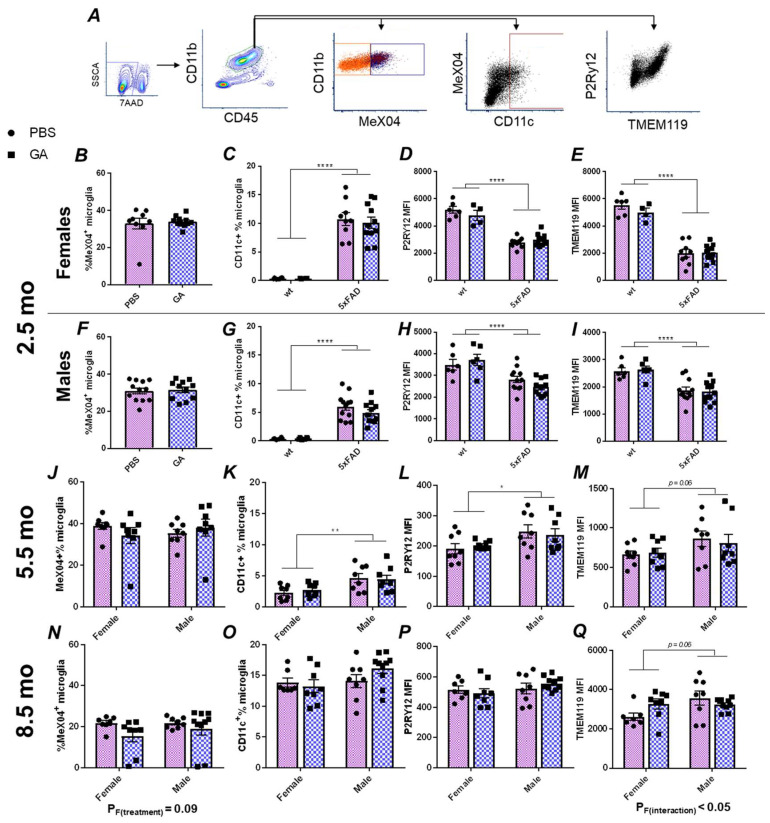
Flow cytometry analysis of hippocampal microglia reveals no major changes following GA treatment of 5xFAD mice. Gating strategy (**A**). Microglia were gated as CD45^int^CD11b^+^ events (green hexagon). MeX04^+^ (dark blue box) and negative (orange box), as well as CD11c^+^ (dark red box) populations are readily identifiable. GA treatment had no effect on MeX04^+^ percentage of microglia in female or male mice in the 2.5 (**B**,**F**), 5.5 (**J**), or 8.5-month-old (**N**) cohorts. Quantification of percentage of CD11c^+^ microglia showed no significant changes with GA treatment in 2.5 (**C**,**G**), 5.5 (**K**), or 8.5-month-old (**O**) cohorts. Quantification of microglial P2Ry12 MFI (**D**,**H**,**L**,**P**) or TMEM119 MFI (**E**,**I**,**M**,**Q**) revealed no significant effect of GA treatment in female or male mice in any of the cohorts. As expected, wt mice had less CD11c^+^ microglia (**C**,**G**), and decreased levels of TMEM119 (**D**,**H**) and P2Ry12 (**E**,**I**) compared to 5xFAD mice in the 2.5-month-old cohort. Numerical data represented as mean ± SEM using circles and pink boxes for PBS treatment and squares and blue boxes for GA treatment. *n* = 4–12 animals per group. * *p* < 0.05, ** *p* < 0.01, **** *p* < 0.0001. Two-way ANOVA with post hoc multiple comparisons except for (**B**,**F**) in which Student’s *t*-tests were used.

Based on the extensive literature on GA’s effect on the immune system, we hypothesized that microglia, as the immunocompetent macrophages of the CNS, should respond to GA treatment regardless of the cellular mechanism that allows the communication between the periphery and the CNS. Therefore, we performed flow cytometric characterizations of microglial plaque uptake, microglial expression of CD11c, and microglial expression of the homeostatic markers TMEM119 and P2Ry12 (Figure 2A) in microglia isolated from the hippocampus.

Within CD45^int^CD11b^+^ microglia, we did not detect any statistically significant changes in the percentage of MeX04^+^ cells in males or females at any of the timepoints at which GA treatment was initiated (Figure 2B,F,J,N). Because CD11c is one of the DAM markers, and earlier reports had demonstrated its expression in microglia following GA treatment [22], we chose to use it as a proxy to quantify the DAM population [23]. We did not find any GA treatment induced changes in the percentage of CD11c^+^ microglia at any of our timepoints (Figure 2C,G,K,O). Lastly, we also did not find any statistically significant changes in the median fluorescence intensities (MFIs) of P2Ry12 or TMEM119 in response to GA treatment in any sex at any of the timepoints examined (Figure 2D,E,H,I,L,M,P,Q).

### 3.3. GA Treatment Does Not Increase DAM Proximal to Plaque

**Figure 3:**
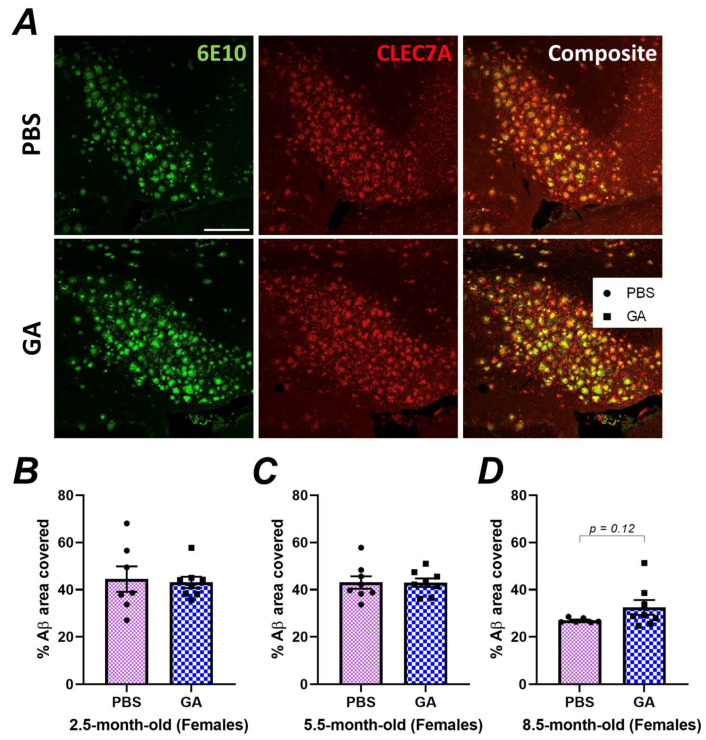
GA treatment does not lead to an increase in CLEC7A^+^ microglia proximal to 6E10^+^ Aβ plaques. Representative 20× images show 6E10 (green), CLEC7A^+^ cells (red), and their composite (**A**). Scale bar represents 150 μm. Quantification of the plaque area covered by CLEC7A^+^ microglia revealed no statistically significant differences between GA- or PBS-treated mice in the 2.5 (**B**), 5.5 (**C**), or 8.5-month-old (**D**) cohorts. *n* = 6–7 animals per group. Student’s *t*-tests.

Our immunohistochemistry experiments indicated neuroanatomic differences in plaque load in response to GA, especially in the subiculum of female 5xFAD mice (Figure 1). However, flow cytometric quantification of DAM, which may impact plaque levels, was performed on microglia isolated from the entire hippocampus, which might have masked subtle differences in hippocampal substructures. To analyze the microglial phenotype specifically in the subiculum of female 5xFAD, we co-stained 6E10^+^ Aβ plaques with CLEC7A (Figure 3A), which is a well-established marker for DAM microglia [34,35]. Quantification of the colocalization of CLEC7A^+^ microglia proximal to Aβ plaques did not reveal a statistically significant difference between PBS- and GA-treated mice at any of the timepoints (Figure 3B–D). These data support the hypothesis that GA treatment does not increase DAM found within the vicinity of 6E10^+^ Aβ plaques.

## 4. Discussion

Due to its ability to induce type 2 responses and its excellent safety profile, GA has been one of the candidate therapeutics of interest for repurposed use in AD [10,36]. Various reports, including some from our research group, have demonstrated beneficial disease modifying effects of GA in AD-like mouse models [25,26,27,28,29]. However, in this study, we failed to identify a clear impact of GA on reducing Aβ plaques. While we saw a trend towards decreased plaque when the GA treatment was initiated at 5.5 months of age, we saw significantly increased plaque burden when GA treatment was begun at 8.5 months of age as assessed by 6E10 staining (Figure 1). Furthermore, this effect was specific to females as there were no changes in the plaque load in males (Figure 1). These effects were not observed with MeX04 staining, which may be due to the fact that MeX04 staining does not detect soluble Aβ peptides, or the amyloid precursor protein (APP) or its fragments.

Although the impact of GA in the APP/PS1 model of amyloidosis has been reported multiple times, currently there is only one other study of GA use in 5xFAD mice [28]. In stark contrast to our data, Baruch et al. showed that in addition to improving behavioral performance in the water maze, GA decreased 6E10^+^ Aβ plaque area with 4 weeks of treatment beginning at 7 months of age [28]. They also showed that 4 weeks of GA treatment beginning at 5 months of age can significantly decrease plaque in the DG compared to 6-month-old mice that have not undergone any treatment [28]. In our experiments, we chose to use the previously published 2-month-long GA treatment paradigm [27,29] as opposed to the 4-week treatment used in Baruch et al. since 4 weeks of GA treatment had no effect on the plaque burden in our previous experiments in 3xTg mice, but 8 weeks of GA treatment did have an effect [27]. Furthermore, in contrast to Baruch et al., we split our analyses by sex because there were significant effects of sex in our 2.5-month-old cohort (Figure 1). Due to these discrepant results, our data strongly recommend the use of caution when repurposing the use of GA to advanced AD pathology. Lastly, our data suggest that GA is likely more effective in females during intermediate levels of AD pathology, since GA had no effect on male mice.

The mechanism of GA is unclear. In the brain, within the context of AD, GA was shown to induce expression of CD11c in microglia [22]. Studies a decade later identified that all DAM cells in 5xFAD mice were CD11c^+^ and that CD11c^+^ microglia display highly overlapping gene expression signatures with the DAM transcriptional profile [23,37]. Furthermore, since DAM are induced in response to Aβ, it is not surprising to see higher MeX04 positivity in the identified CD11c^+^ population (Figure 2). Altogether, these observations raise the hypothesis that GA may be inducing DAM. In flow cytometric characterization of microglia following GA treatment, we saw no significant changes in CD11c immunopositivity or any loss in the homeostatic P2Ry12 levels, which is inconsistent with this hypothesis. Furthermore, we did not detect increased DAM around plaques even in hippocampal substructures which had shown decreased plaque load in response to GA treatment (Figure 3). Although our data are consistent with our previous findings of GA’s impact in 3xTg mice, which revealed very minimal changes in microglial gene expression, we acknowledge that testing GA’s impact on DAM with only a handful of markers is limited and future studies should consider single cell sequencing approaches or molecular topography to determine plaque-specific responses.

Overall, we provide evidence that GA is more effective in female 5xFAD mice than in males. In contrast to previously published literature, we show that GA may be detrimental to plaque pathology if administrated late during disease progression. Our data do not support a strong microglial impact of GA. We do not identify any major changes in microglial levels of CD11c, TMEM119, or P2Ry12, but encourage future investigations of the microglial response to GA with higher resolution techniques, such as single cell RNAseq. We argue that caution must be exercised when attempting to repurpose GA for the treatment of AD.

## Data Availability

The data presented in this study are available on request from the corresponding author.

## Supplementary Materials

The following supporting information can be downloaded at: https://www.mdpi.com/article/10.3390/pharmaceutics15071809/s1, Supplementary Figure S1. Tracking of bodyweights suggests that GA does not negatively impact overall health of the mice. Mice were weighed a day before the initiation of GA treatment and weekly thereafter and we found no GA-induced changes in bodyweight in male or female mice in the 2.5 (A), 5.5 (B), or 8.5-month-old (C) cohorts. Supplementary Figure S2. GA treatment does not change plaque load in CA1. Please refer to Figure 1A for representative images. GA treatment has no significant effect on 6E10^+^ Aβ plaques (A,E,I) or MeX04^+^ plaques (B,F,J) in female or male 5xFAD mice at any of the timepoints investigated. Similarly, GA treatment does not lead to a significant change in LAMP1 immunopositivity (C,G,K). Analysis of Iba1^+^ microglial colocalization with 6E10^+^ Aβ plaques shows no significant effect of GA treatment at the 2.5 (D), 5.5 (H), or 8.5-month (L) timepoints but shows a trend towards interaction of sex and GA treatment in the 5.5-month-old cohort (H) and a significant interaction of sex and GA treatment in the 8.5-month-old cohort (L). Numerical data represented as mean ± SEM using circles and pink boxes for PBS treatment and squares and blue boxes for GA treatment. *n* = 7–10 animals per group. * *p* < 0.05, ** *p* < 0.01, *** *p* < 0.001. Two-way ANOVA with post hoc multiple comparisons. Supplementary Figure S3. GA treatment does not change plaque load in DG. Please refer to Figure 1A for representative images. GA treatment has no significant effect on 6E10^+^ Aβ plaques (A,E,I) or MeX04^+^ plaques (B,F,J) in female or male 5xFAD mice at any of the timepoints investigated. Similarly, GA treatment does not lead to a significant change in LAMP1 immunopositivity (C,G,K). GA treatment had no significant effect on 6E10^+^ Aβ plaque area covered by Iba1^+^ microglia in the 2.5 (D) and 5.5-month-old (H) cohorts but there was a significant interaction of GA treatment with sex in the 8.5-month-old cohort and GA significantly reduced microglial colocalization with plaque in female mice (L). Numerical data represented as mean ± SEM using circles and pink boxes for PBS treatment and squares and blue boxes for GA treatment. *n* = 7–10 animals per group. * *p* < 0.05, ** *p* < 0.01, *** *p* < 0.001. Two-way ANOVA with post hoc multiple comparisons.

## References

[1] Selkoe D.J., Hardy J. (2016). The amyloid hypothesis of Alzheimer’s disease at 25 years. EMBO Mol. Med..

[2] De Strooper B., Karran E. (2016). The Cellular Phase of Alzheimer’s Disease. Cell.

[3] Hickman S., Izzy S., Sen P., Morsett L., El Khoury J. (2018). Microglia in neurodegeneration. Nat. Neurosci..

[4] Hansen D.V., Hanson J.E., Sheng M. (2018). Microglia in Alzheimer’s disease. J. Cell Biol..

[5] Kawahara K., Suenobu M., Yoshida A., Koga K., Hyodo A., Ohtsuka H., Kuniyasu A., Tamamaki N., Sugimoto Y., Nakayama H. (2012). Intracerebral microinjection of interleukin-4/interleukin-13 reduces beta-amyloid accumulation in the ipsilateral side and improves cognitive deficits in young amyloid precursor protein 23 mice. Neuroscience.

[6] Kiyota T., Okuyama S., Swan R.J., Jacobsen M.T., Gendelman H.E., Ikezu T. (2010). CNS expression of anti-inflammatory cytokine interleukin-4 attenuates Alzheimer’s disease-like pathogenesis in APP+PS1 bigenic mice. FASEB J..

[7] Shaftel S.S., Kyrkanides S., Olschowka J.A., Jen-nie H.M., Johnson R.E., O’Banion M.K. (2007). Sustained hippocampal IL-1 beta overexpression mediates chronic neuroinflammation and ameliorates Alzheimer plaque pathology. J. Clin. Investig..

[8] Ghosh S., Wu M.D., Shaftel S.S., Kyrkanides S., LaFerla F.M., Olschowka J.A., O’Banion M.K. (2013). Sustained interleukin-1beta overexpression exacerbates tau pathology despite reduced amyloid burden in an Alzheimer’s mouse model. J. Neurosci..

[9] Cherry J.D., Olschowka J.A., O’Banion M.K. (2015). Arginase 1+ microglia reduce Abeta plaque deposition during IL-1beta-dependent neuroinflammation. J. Neuroinflamm..

[10] Cherry J.D., Olschowka J.A., O’Banion M.K. (2014). Neuroinflammation and M2 microglia: The good, the bad, and the inflamed. J. Neuroinflamm..

[11] Paolicelli R.C., Sierra A., Stevens B., Tremblay M.-E., Aguzzi A., Ajami B., Amit I., Audinat E., Bechmann I., Bennett M. (2022). Microglia states and nomenclature: A field at its crossroads. Neuron.

[12] Kyrkanides S., Tallents R.H., Miller J.-N.H., Olschowka M.E., Johnson R., Yang M., Olschowka J.A., Brouxhon S.M., O’Banion M.K. (2011). Osteoarthritis accelerates and exacerbates Alzheimer’s disease pathology in mice. J. Neuroinflamm..

[13] Lee J.W., Lee Y.K., Yuk D.Y., Choi D.Y., Ban S.B., Oh K.W., Hong J.T. (2008). Neuro-inflammation induced by lipopolysaccharide causes cognitive impairment through enhancement of beta-amyloid generation. J. Neuroinflamm..

[14] Lee D.C., Rizer J., Selenica M.-L.B., Reid P., Kraft C., Johnson A., Blair L., Gordon M.N., Dickey C.A., Morgan D. (2010). LPS- induced inflammation exacerbates phospho-tau pathology in rTg4510 mice. J. Neuroinflamm..

[15] Arnon R., Aharoni R. (2019). Glatiramer Acetate: From Bench to Bed and Back. Isr. Med. Assoc. J..

[16] Teitelbaum D., Meshorer A., Hirshfeld T., Arnon R., Sela M. (1971). Suppression of experimental allergic encephalomyelitis by a synthetic polypeptide. Eur. J. Immunol..

[17] Aharoni R. (2013). The mechanism of action of glatiramer acetate in multiple sclerosis and beyond. Autoimmun. Rev..

[18] Aharoni R., Eilam R., Stock A., Vainshtein A., Shezen E., Gal H., Friedman N., Arnon R. (2010). Glatiramer acetate reduces Th-17 inflammation and induces regulatory T-cells in the CNS of mice with relapsing-remitting or chronic EAE. J. Neuroimmunol..

[19] Aharoni R., Kayhan B., Eilam R., Sela M., Arnon R. (2003). Glatiramer acetate-specific T cells in the brain express T helper 2/3 cytokines and brain-derived neurotrophic factor in situ. Proc. Natl. Acad. Sci. USA.

[20] Weber M.S., Prod’Homme T., Youssef S., Dunn S.E., Rundle C.D., Lee L., Patarroyo J.C., Stüve O., Sobel R.A., Steinman L. (2007). Type II monocytes modulate T cell-mediated central nervous system autoimmune disease. Nat. Med..

[21] Prod’homme T., Zamvil S.S. (2019). The Evolving Mechanisms of Action of Glatiramer Acetate. Cold Spring Harb. Perspect. Med..

[22] Butovsky O., Koronyo-Hamaoui M., Kunis G., Ophir E., Landa G., Cohen H., Schwartz M. (2006). Glatiramer acetate fights against Alzheimer’s disease by inducing dendritic-like microglia expressing insulin-like growth factor 1. Proc. Natl. Acad. Sci. USA.

[23] Keren-Shaul H., Spinrad A., Weiner A., Matcovitch-Natan O., Dvir-Szternfeld R., Ulland T.K., David E., Baruch K., Lara-Astaiso D., Toth B. (2017). A Unique Microglia Type Associated with Restricting Development of Alzheimer’s Disease. Cell.

[24] Hu Y., Fryatt G.L., Ghorbani M., Obst J., Menassa D.A., Martin-Estebane M., Muntslag T.A.O., Olmos-Alonso A., Guerrero-Carrasco M., Thomas D. (2021). Replicative senescence dictates the emergence of disease-associated microglia and contributes to Abeta pathology. Cell Rep..

[25] Doustar J., Rentsendorj A., Torbati T., Regis G.C., Fuchs D., Sheyn J., Mirzaei N., Graham S.L., Shah P.K., Mastali M. (2020). Parallels between retinal and brain pathology and response to immunotherapy in old, late-stage Alzheimer’s disease mouse models. Aging Cell.

[26] Rentsendorj A., Sheyn J., Fuchs D.T., Daley D., Salumbides B.C., Schubloom H.E., Hart N.J., Li S., Hayden E.Y., Teplow D.B. (2018). A novel role for osteopontin in macrophage-mediated amyloid-beta clearance in Alzheimer’s models. Brain Behav. Immun..

[27] Dionisio-Santos D.A., Karaahmet B., Belcher E.K., Owlett L.D., Trojanczyk L.A., Olschowka J.A., O’banion M.K. (2021). Evaluating Effects of Glatiramer Acetate Treatment on Amyloid Deposition and Tau Phosphorylation in the 3xTg Mouse Model of Alzheimer’s Disease. Front. Neurosci..

[28] Baruch K., Rosenzweig N., Kertser A., Deczkowska A., Sharif A.M., Spinrad A., Tsitsou-Kampeli A., Sarel A., Cahalon L., Schwartz M. (2015). Breaking immune tolerance by targeting Foxp3(+) regulatory T cells mitigates Alzheimer’s disease pathology. Nat. Commun..

[29] Koronyo Y., Salumbides B.C., Sheyn J., Pelissier L., Li S., Ljubimov V., Moyseyev M., Daley D., Fuchs D.-T., Pham M. (2015). Therapeutic effects of glatiramer acetate and grafted CD115(+) monocytes in a mouse model of Alzheimer’s disease. Brain.

[30] Oakley H., Cole S.L., Logan S., Maus E., Shao P., Craft J., Guillozet-Bongaarts A.L., Ohno M., Disterhoft J., Van Eldik L. (2006). Intraneuronal beta-amyloid aggregates, neurodegeneration, and neuron loss in transgenic mice with five familial Alzheimer’s disease mutations: Potential factors in amyloid plaque formation. J. Neurosci..

[31] Oblak A.L., Lin P.B., Kotredes K.P., Pandey R.S., Garceau D., Williams H.M., Uyar A., O’rourke R., O’rourke S., Ingraham C. (2021). Comprehensive Evaluation of the 5XFAD Mouse Model for Preclinical Testing Applications: A MODEL-AD Study. Front. Aging Neurosci..

[32] Rivera-Escalera F., Pinney J.J., Owlett L., Ahmed H., Thakar J., Olschowka J.A., Elliott M.R., O’Banion M.K. (2019). IL-1beta-driven amyloid plaque clearance is associated with an expansion of transcriptionally reprogrammed microglia. J. Neuroinflamm..

[33] Klunk W.E., Bacskai B.J., Mathis C.A., Kajdasz S.T., McLellan M.E., Frosch M.P., Debnath M.L., Holt D.P., Wang Y., Hyman B.T. (2002). Imaging Abeta plaques in living transgenic mice with multiphoton microscopy and methoxy-X04, a systemically administered Congo red derivative. J. Neuropathol. Exp. Neurol..

[34] Krasemann S., Madore C., Cialic R., Baufeld C., Calcagno N., El Fatimy R., Beckers L., O’Loughlin E., Xu Y., Fanek Z. (2017). The TREM2-APOE Pathway Drives the Transcriptional Phenotype of Dysfunctional Microglia in Neurodegenerative Diseases. Immunity.

[35] Deczkowska A., Keren-Shaul H., Weiner A., Colonna M., Schwartz M., Amit I. (2018). Disease-Associated Microglia: A Universal Immune Sensor of Neurodegeneration. Cell.

[36] Kasindi A., Fuchs D.-T., Koronyo Y., Rentsendorj A., Black K.L., Koronyo-Hamaoui M. (2022). Glatiramer Acetate Immunomodulation: Evidence of Neuroprotection and Cognitive Preservation. Cells.

[37] Kamphuis W., Kooijman L., Schetters S., Orre M., Hol E.M. (2016). Transcriptional profiling of CD11c-positive microglia accumulating around amyloid plaques in a mouse model for Alzheimer’s disease. Biochim. Biophys. Acta.

