# Supplementary material for: Inconsistent Effects of Glatiramer Acetate Treatment in the 5xFAD Mouse Model of Alzheimer’s Disease
Source: Pharmaceutics. 2023 Jun 24;15(7):1809. doi: 10.3390/pharmaceutics15071809 (PMC10383120; doi:10.3390/pharmaceutics15071809)
Supplement: Supplementary file 1 [file pharmaceutics-15-01809-s001.zip › pharmaceutics-2455630-supplementary.pdf]

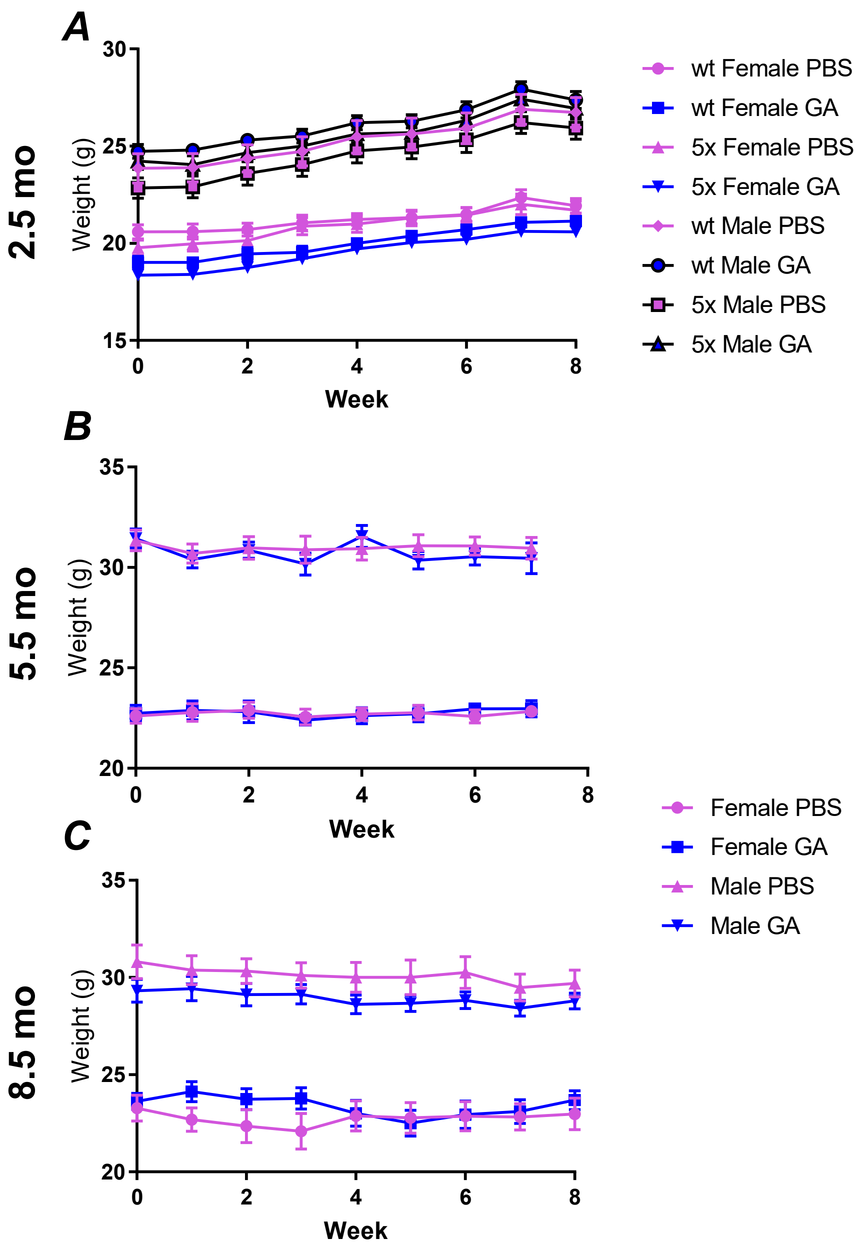

Supplementary Figure S1. Tracking of bodyweights suggests that GA does not negatively impact overall health of the mice. Mice were weighed a day before the initiation of GA treatment and weekly thereafter and we found no GA-induced changes in bodyweight in male or female mice in the 2.5 (A), 5.5 (B), or 8.5-month-old (C) cohorts.

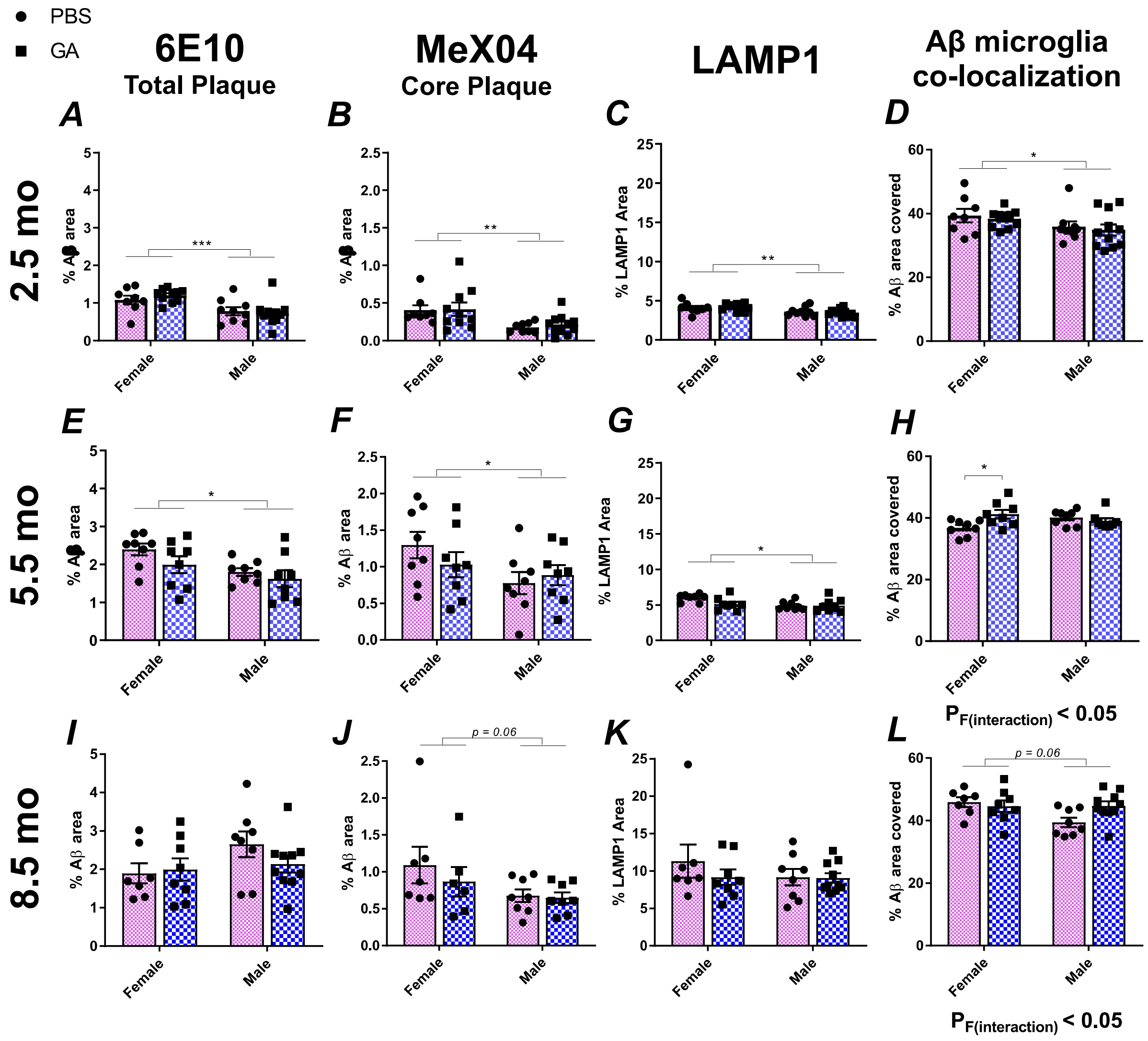

Supplementary Figure S2. GA treatment does not change plaque load in CA1. Please refer to Figure 1A for representative images. GA treatment has no significant effect on 6E10⁺ Aβ plaques (A,E,I) or MeX04⁺ plaques (B,F,J) in female or male 5xFAD mice at any of the timepoints investigated. Similarly, GA treatment does not lead to a significant change in LAMP1 immunopositivity (C,G,K). Analysis of Iba1⁺ microglial colocalization with 6E10⁺ Aβ plaques shows no significant effect of GA treatment at the 2.5 (D), 5.5 (H), or 8.5-month (L) timepoints but shows a trend towards interaction of sex and GA treatment in the 5.5-month-old cohort (H) and a significant interaction of sex and GA treatment in the 8.5-month-old cohort (L). Numerical data represented as mean ± SEM using circles and pink boxes for PBS treatment and squares and blue boxes for GA treatment. *n* = 7–10 animals per group. * $p < 0.05$, ** $p < 0.01$, *** $p < 0.001$. Two-way ANOVA with post hoc multiple comparisons.

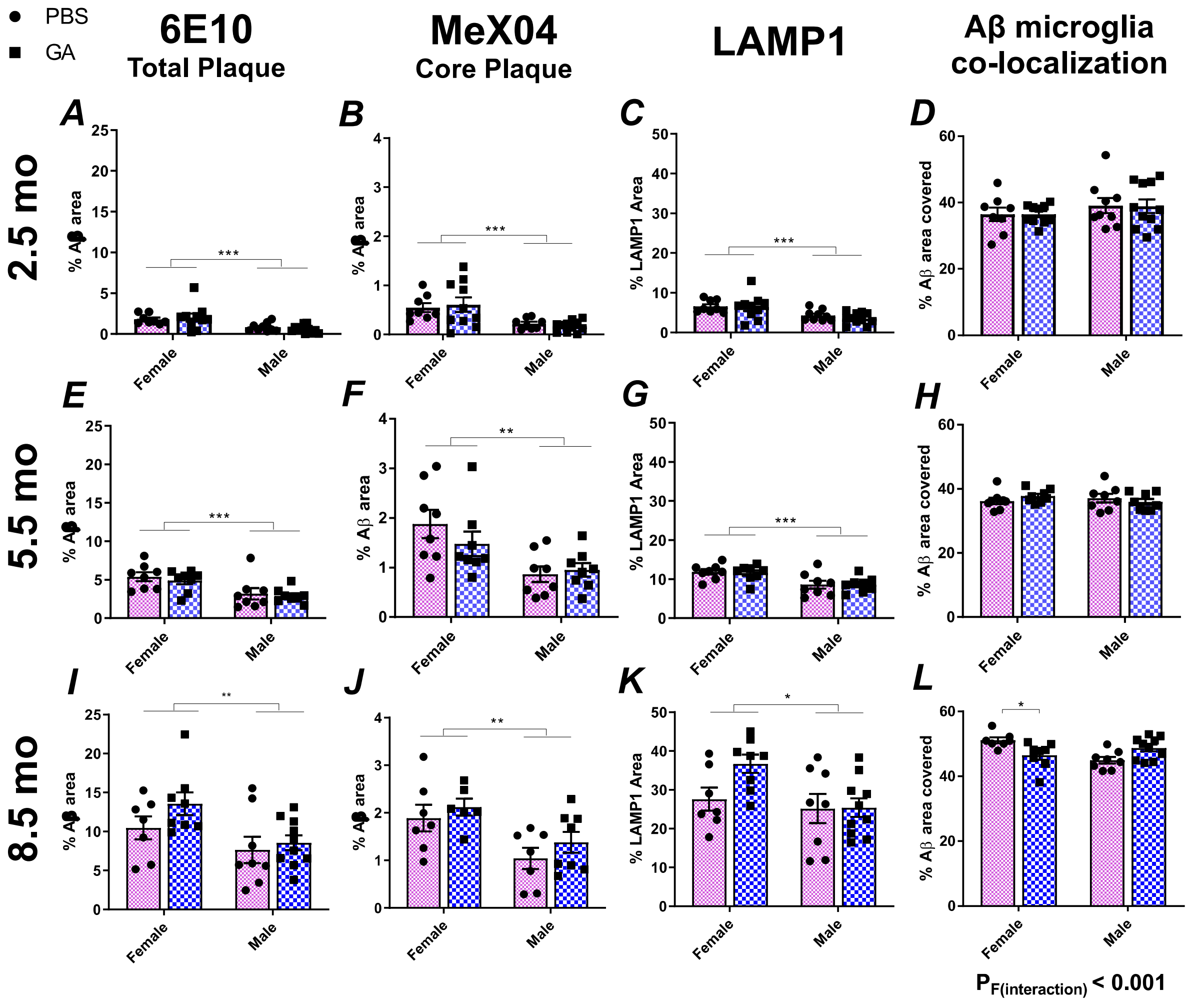

Supplementary Figure S3. GA treatment does not change plaque load in DG. Please refer to Figure 1A for representative images. GA treatment has no significant effect on 6E10⁺ Aβ plaques (A,E,I) or MeX04⁺ plaques (B,F,J) in female or male 5xFAD mice at any of the timepoints investigated. Similarly, GA treatment does not lead to a significant change in LAMP1 immunopositivity (C,G,K). GA treatment had no significant effect on 6E10⁺ Aβ plaque area covered by Iba1⁺ microglia in the 2.5 (D) and 5.5-month-old (H) cohorts but there was a significant interaction of GA treatment with sex in the 8.5-month-old cohort and GA significantly reduced microglial colocalization with plaque in female mice (L). Numerical data represented as mean ± SEM using circles and pink boxes for PBS treatment and squares and blue boxes for GA treatment. n = 7–10 animals per group. * $p < 0.05$, ** $p < 0.01$, *** $p < 0.001$. Two-way ANOVA with post hoc multiple comparisons.
